# Independent association of PCSK9 with platelet reactivity in subjects without statin or antiplatelet agents

**DOI:** 10.3389/fcvm.2022.934914

**Published:** 2022-10-17

**Authors:** Shuai Wang, Di Fu, Huixing Liu, Daoquan Peng

**Affiliations:** Department of Cardiovascular Medicine, Second Xiangya Hospital of Central South University, Changsha, China

**Keywords:** proprotein convertase subtilisin/kexin type 9, platelet aggregation, ADP, cross-sectional study, impedance aggregometry

## Abstract

**Background and aims:**

Proprotein convertase subtilisin/kexin type 9 (PCSK9) levels could predict cardiovascular event in patients with well-controlled LDL-C levels, suggesting an LDL-independent mechanism of PCSK9 on the cardiovascular system. Accumulating evidence suggests PCSK9 might be associated with increased platelet reactivity. This study aimed to assess the relationship between PCSK9 levels and platelet reactivity in subjects not taking statins or antiplatelet agents.

**Methods:**

A cross-sectional study was conducted to investigate the independent contribution of PCSK9 to platelet activity by controlling for the potential confounding factors. The study population included 89 subjects from a health examination centre who underwent routine annual health check-ups or had an examination before a selective operation. Subjects taking statins or antiplatelet agents were excluded. Adenosine diphosphate (ADP)-induced platelet aggregation was determined by PL-11 platelet analyzer using impedance aggregometry and plasma PCSK9 levels were determined using an ELISA. Serum Lipid profile was assessed by measuring the concentration of total cholesterol (TC), high-density lipoprotein cholesterol (HDL-C), and triglyceride (TG), with low-density lipoprotein cholesterol (LDL-C) being directly measured using enzymatic techniques. The association between PCSK9 and platelet reactivity was investigated.

**Results:**

The study subjects were composed of 53 males and 36 females with an average age of 55 (±11) years old. The univariate correlation analysis showed significant correlation between ADP-induced maximal aggregation rate (MAR) and PCSK9 (*r* = 0.55, *p* < 0.001) as well as TC (*r* = 0.23, *p* = 0.028), LDL-C (*r* = 0.27, *p* < 0.001), and PLT (*r* = 0.31, *p* = 0.005). Being male (41.2% vs. 46.6, *p* = 0.04) and smoking (37.4 vs. 46.2%, *p* = 0.016) were associated with lower ADP-induced MAR than being female and non-smoking. However, there is no correlation between PCSK9 and AA-induced platelet maximal aggregation rate (*r* = 0.17, *p* = 0.12). Multiple regression analysis suggested that PCSK9 contributed independently to ADP-induced maximal aggregation rate (β = 0.08, *p* = 0.004) after controlling for the effect of TC, LDL-C, PLT, being male, and smoking.

**Conclusions:**

PCSK9 is positively associated with platelet reactivity, which may partly account for the beneficial effect of PCSK9 inhibition in reducing the risk of major adverse cardiovascular events after acute coronary syndrome (ACS).

## Introduction

Atherosclerotic cardiovascular disease (CVD) is a major cause of disease burden. Platelets have an important role in coronary thrombosis pathogenesis and atherogenesis.

Studies have shown that platelet activity varies greatly among individuals. It could explain the variability in the risk for CVD (1–4). Prior clinical studies found an association between platelet activity and incident cardiovascular morbidity and mortality (5, 6). Hypercholesterolemia and its induced reactive oxygen species production can activate platelets (7–9). However, the molecules through which platelets become hyperactive remain not fully understood.

Proprotein Convertase Subtilisin/Kexin Type 9 (PCSK9), mainly synthesized by the liver, kidney, and small intestine, bind and inhibit low density lipoprotein receptor (LDLR) recircularization by promoting its degradation in the lysosomes and consequently increase low density lipoprotein (LDL) particles in the circulation (10). Regulation of cholesterol-rich LDL level is not the only role that PCSK9 has in atherosclerosis pathogenesis. In prospective cohort studies, plasma PCSK9 level was correlated with enhanced atherosclerosis progression and elevated probability of future cardiovascular events independently of LDL plasma levels, suggesting alternative roles for PCSK9 in the pathogenesis of atherosclerosis (11, 12).

A relationship between PCSK9 plasma levels and total number of circulating platelets has been reported in patients with stable coronary artery disease (13). A strong correlation between PCSK9 levels and platelet reactivity was also revealed in patients with recent acute coronary syndromes who underwent coronary intervention and received P2Y_12_ inhibitors (14). However, statin use could increase PCSK9 levels and antiplatelet drugs could affect platelet activity in CAD patients (15, 16). In another study, human recombinant PCSK9 added to healthy human plasma significantly increased platelet aggregation when stimulated with epinephrine (17). But the concentration of human recombinant PCSK9 used in an *in-vitro* study was much higher than the physiological concentration in humans. Therefore, the naive correlation between plasma PCSK9 and platelet reactivity in subjects without lipid lowering or antiplatelet drugs is not known.

In the present study, we revealed a correlation between plasma PCSK9 and platelet reactivity when stimulated by agonist adenosine diphosphate (ADP) *in vitro* in healthy subjects without lipid-lowering or antiplatelet drugs. The ADP-induced maximal aggregation rate of platelets was 15.8% higher in patients with highest tertile PCSK9 value than the patients in the lowest tertile. Additionally, we found that PCSK9 was independently correlated to platelet activity after adjusting for low density lipoprotein cholesterol (LDL-C) and platelet (PLT) count. The results of this study provide another piece of evidence on the correlation between PCSK9 and platelet activity in healthy subjects.

## Methods

### Study population and design

This study is a cross-sectional, single center clinical study. Eighty-nine subjects who underwent routine annual health check-ups or had an examination before a selective operation were enrolled from a health examination center. Inclusion criteria were: 1) aged between 18 and 80 years and 2) obtained signed informed consent. The exclusion criteria were subjects with atherosclerotic cardiovascular disease (ASCVD), malignant tumor, renal dysfunction, liver dysfunction, thyroid disease, autoimmune disease, or coagulation disorders. Subjects who had lipid lowering drugs or antiplatelet drugs in the past 3 months before screening were excluded. Hypertension and diabetes were defined as being present when an individual self-reported a health professional's diagnosis and was using associated drugs. Written informed consent was obtained from each patient included in the study. The study protocol conforms to the ethical guidelines of the 1975 Declaration of Helsinki and the study protocol has been priorly approved by the ethics committee of Second Xiangya Hospital on research on humans.

### Assessment of platelet reactivity

A blood sample was withdrawn after overnight fasting and analyzed for platelet reactivity within 2 h. Whole blood aggregation was determined using PL-11 platelet analyzer (SINNOWA, Nanjing). The system detects the electrical impedance change due to the adhesion and aggregation of platelet on two independent electrode-set surfaces. Sodium citrate was used as an anticoagulant; adenosine diphosphate and arachidonic acid were used as agonists. A 1:9 dilution of whole blood anticoagulated with sodium citrate and 0.9% NaCl was stirred at 25°C. ADP 5 0μmol/L and arachidonic acid (AA) 2 mg/mL were added.

### Assessment of PCSK9 serum levels

Blood samples were collected after overnight fasting and the samples were stored at −80°C until analysis. Plasma PCSK9 concentration was determined by a sandwich enzyme-linked immunosorbent assay (ELISA). Commercial PCSK9 (Quantikine ELISA, R&D systems Inc.) ELISA kits were used to quantify the concentrations of them.

### Statistical analysis

For clinical data, continuous data are expressed as mean and standard deviation for normally distributed data or median and interquartile range for non-Gaussian data distribution. For comparison of variables between different groups of tertile PCSK9 values, one-way ANOVA test was used for normally distributed data, with LSD performed for multiple comparisons. Kruskal-Wallis test was used for non-Gaussian distributed data. The distribution of data was examined with the Kolmogorov-Smirnov test. Categorical variables are presented as percentages of subjects and were compared using Pearson *X*^2^ or Fisher's exact tests, as appropriate. The correlation between PCSK9, other lipid parameters, PLT, and platelet activity was evaluated by Spearman's rank test. Continuous PCSK9 levels were categorized into tertiles of equal size to assess the association with ADP-induced maximal aggregation rate (MAR). Platelet reactivity above mean value was classified as higher. Multivariate linear regression was used to assess the association between PCSK9 levels and ADP-induced MAR. Unstandardized *B* and *p*-values were used to present results of the linear regression model. Adjusted R square and *p*-values were used to present for goodness of fit of the multivariate linear regression. All tests were two-sided; a *p* < 0.05 was considered statistically significant. Calculations were performed using SPSS version 26.0 (IBM Corporation, Chicago, USA).

## Results

### Study population

Between 2020 and 2021, 89 subjects, including 53 male and 36 female with an average age of 55 (±11) years, were recruited at Second Xiangya hospital. All subjects had not taken statins or antiplatelet agents before. Baseline clinical characteristics, comorbidities, and laboratory tests of participants according to tertile of PCSK9 are summarized in Supplementary Table 1.

### Factors correlate with platelet reactivity

**Figure 1:**
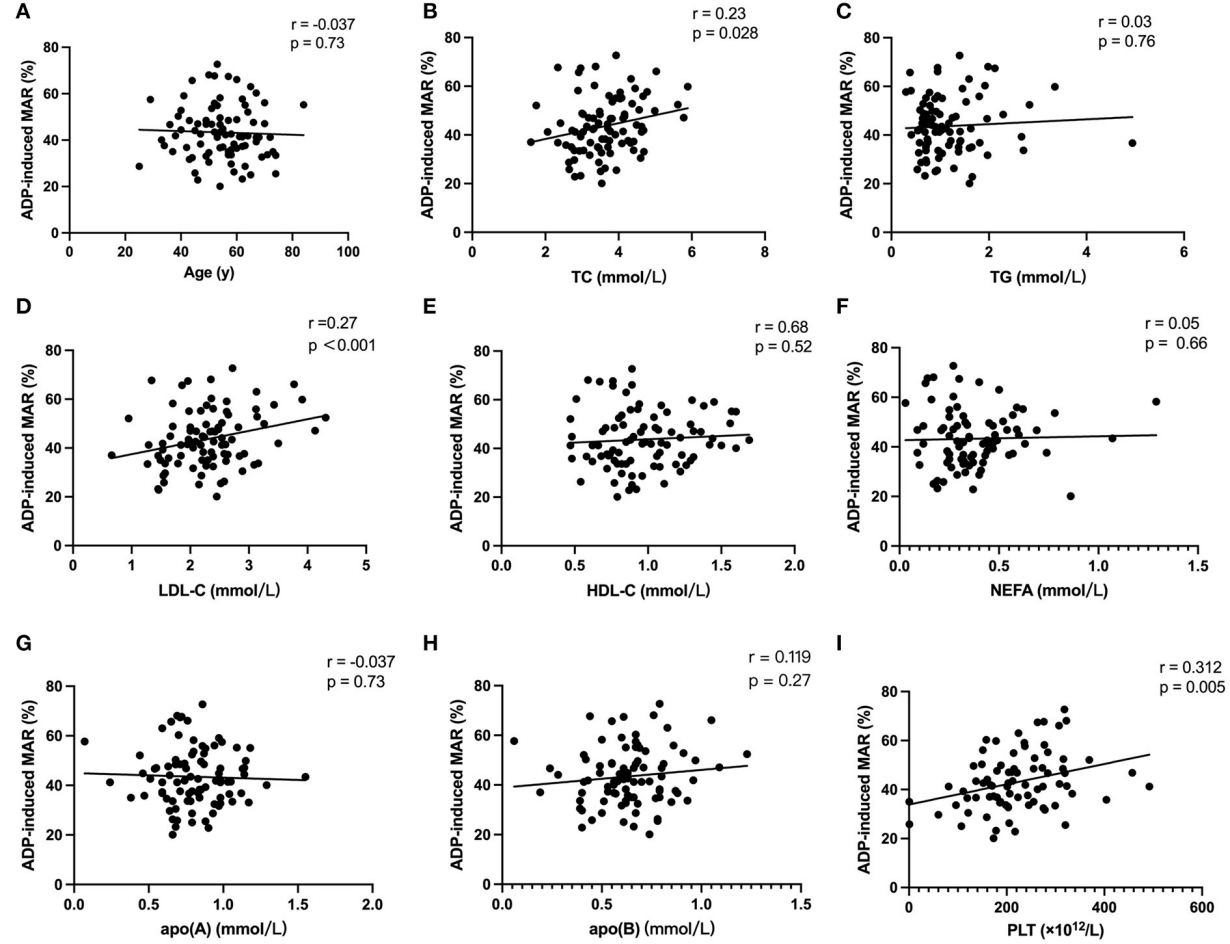
Correlation analysis. Univariate linear correlation analysis of subject characteristics including age **(A)**, TC **(B)**, TG **(C)**, LDL-C **(D)**, HDL-C **(E)**, NEFA **(F)**, apoA **(G)**, apoB **(H)**, and PLT **(I)** with ADP-induced platelet maximal aggregation rate (MAR).

Correlation analysis including all characteristic parameters revealed a significant correlation of ADP-induced MAR with total cholesterol (TC) (*r* = 0.23, *p* = 0.028), LDL-C (*r* = 0.27, *p* <0.001), and PLT (*r* = 0.312, *p* = 0.005). No significant correlation was found between ADP-induced MAR with age and other lipid parameters including plasma triglyceride (TG), high-density lipoprotein cholesterol (HDL-C), nonesterified fatty acid (NEFA), apolipoprotein A, [apo(A)], and apolipoprotein B [apo(B)] (Figure 1).

**Figure 2:**
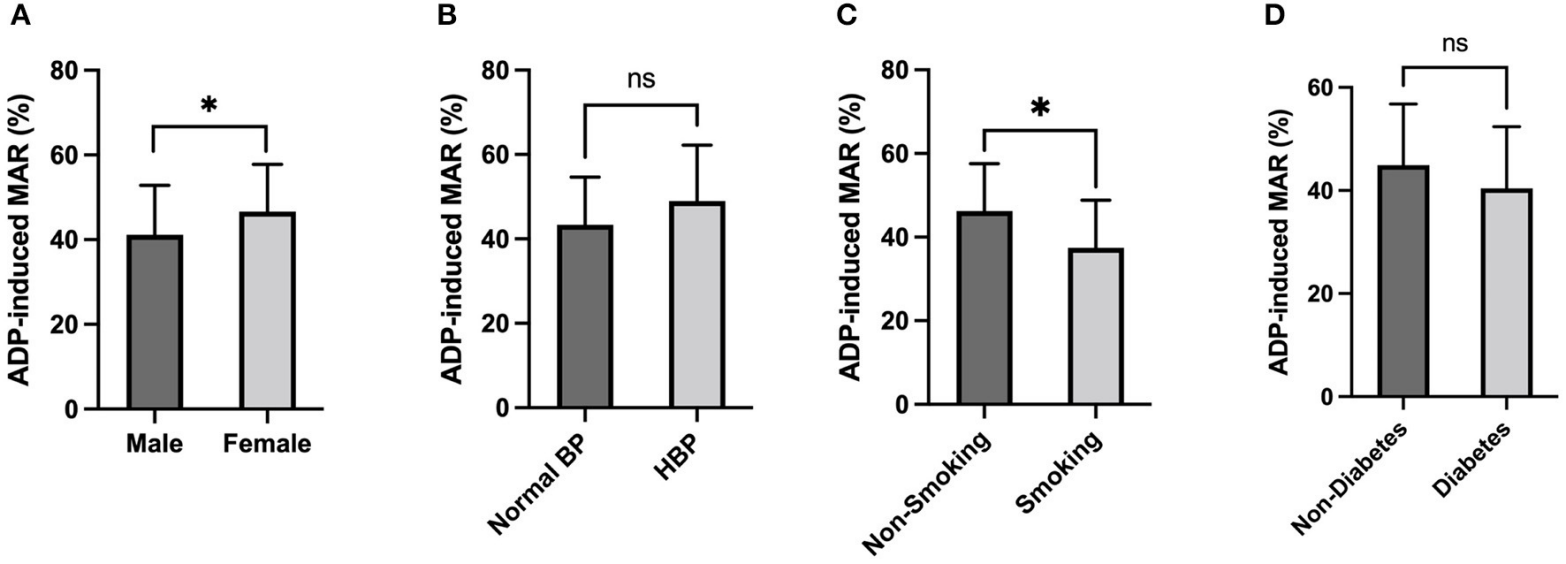
ADP-induced platelet aggregation stratified by Sex and Comorbidities. ADP-induced platelet maximal aggregation rate (MAR) in healthy subjects stratified by Sex **(A)**, Hypertension **(B)**, Smoking **(C)**, and Diabetes **(D)**. *: *P* < 0.0332, ns: *P*>0.1234.

A significantly higher ADP-induced MAR was also observed in females compared to males (46.6 ± 11.1% vs. 41.2 ± 11.7%, *p* = 0.039) and in non-smoking subjects (46.2 ± 11.4% vs. 37.4 ± 11.4%, *p* = 0.016) in comparison to smokers. No significant difference in ADP-induced MAR was found in subjects with or without hypertension and diabetes (Figure 2).

### Plasma total cholesterol, low density lipoprotein cholesterol, and platelet reactivity

**Figure 3:**
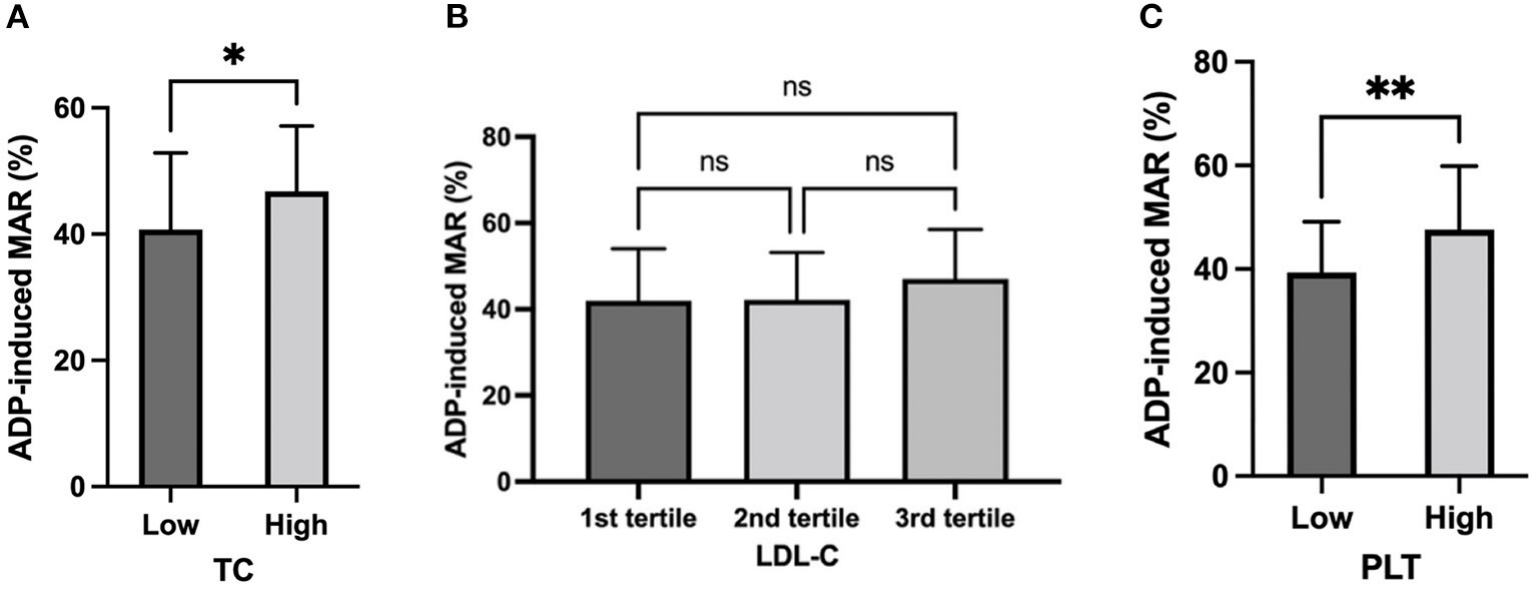
ADP-induced platelet aggregation stratified by TC, LDL-C, and PLT. ADP-induced platelet maximal aggregation rate (MAR) in healthy subjects stratified by TC **(A)**, LDL-C **(B)**, and PLT **(C)**. *: *P* < 0.033, **: *P* < 0.002, ns: *P*>0.1234.

**Figure 4:**
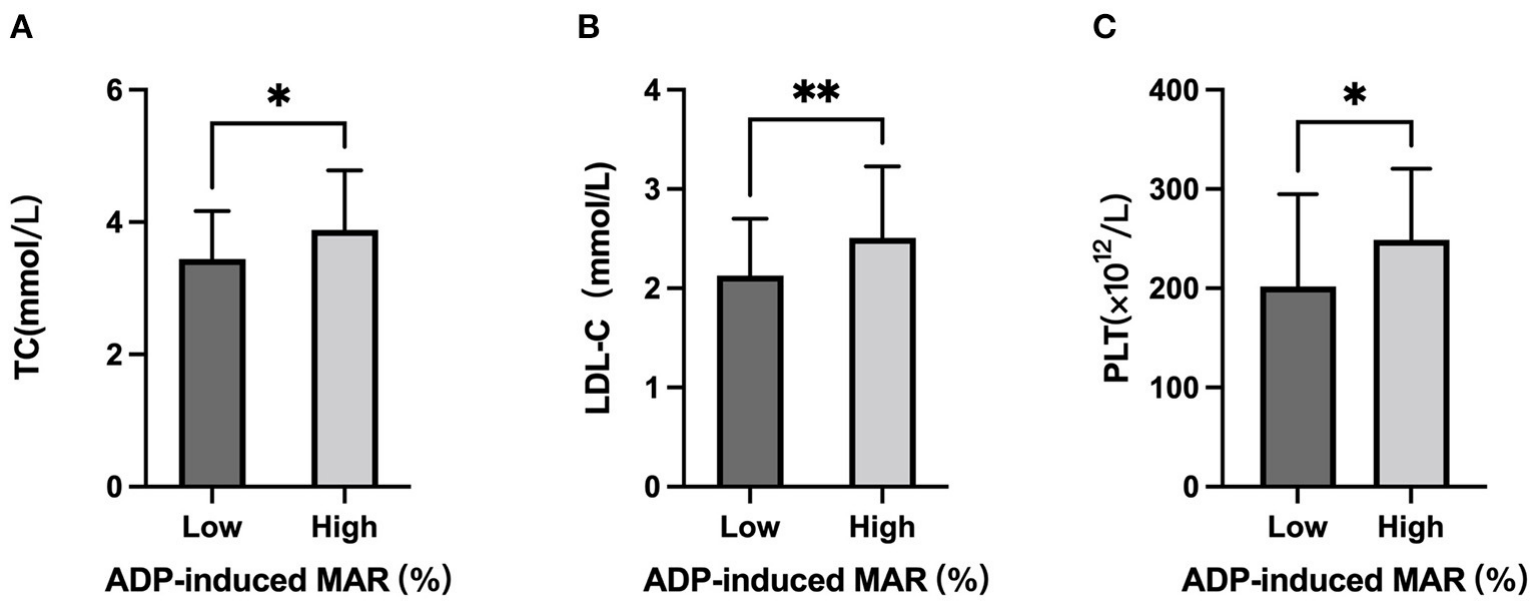
TC, LDL-C, and PLT stratified by low and high ADP-induced platelet aggregation rate. Comparison of **(A)** Serum TC level, **(B)** serum LDL-C level, and **(C)** PLT in subjects with ADP-induced maximal platelet aggregation rate (MAR) lower and higher than mean value. *: *P* < 0.033, **: *P* < 0.002, ns: *P*>0.1234.

In accordance with correlation analysis, a significantly higher ADP-induced MAR was observed in subjects with higher TC level (TC≥3.64 mmol/L:46.8 ± 10.3% vs. TC<3.64 mmol/L: 40.7 ± 12.1%, *p* = 0.013; Figure 3A). On the other hand, subjects with ADP-induced MAR higher than the mean value had significantly higher TC level (3.88 ± 0.90 mmol/L vs. 3.44 ± 0.72 mmol/L, *p* = 0.013; Figure 4A).

Although no significant difference of ADP-induced MAR was observed between tertile LDL-C groups or between high vs. low LDL-C groups according to mean value (Figure 3B), those who had higher platelet reactivity had significantly higher LDL-C levels (2.51 ± 0.72 mmol/L vs. 2.13 ± 0.57 mmol/L; Figure 4B).

### Platelet count and platelet reactivity

In line with the correlation analysis, ADP-induced MAR was significantly higher in subjects with higher PLT (PLT≥220.6 × 10^12^/L: 47.6 ± 12.3% vs. PLT<220.6 × 10^12^/L: 39.4 ± 9.8%; Figure 3C). Vise versa, those who had higher platelet reactivity also had significantly higher PLT [ADP-induced MAR≥mean (43.7%): 248.4 ± 71.9 × 10^12^/L vs. ADP-induced MAR<43.7%: 201.7 ± 93.0 × 10^12^/L] (Figure 4C).

### Plasma PCSK9 concentration and platelet reactivity

Analysis of the correlation between plasma PCSK9 concentration and baseline characteristics revealed that PCSK9 concentration was not correlated with age. Except for LDL-C (*r* = 0.23, *p* = 0.03), PCSK9 concentration was not significantly correlated with other lipid parameters. A higher plasma PCSK9 was observed in subjects with hypertension (246.1 ± 53.8 vs. 207.6 ± 57.0, *p* = 0.03) and in non-smoking subjects (221.8 ± 58.6 vs. 185.8 ± 43.3, *p* = 0.043). No significant difference in PCSK9 level was observed between females and males, nor in subjects with and without diabetes (Supplementary Figure 1).

**Figure 5:**
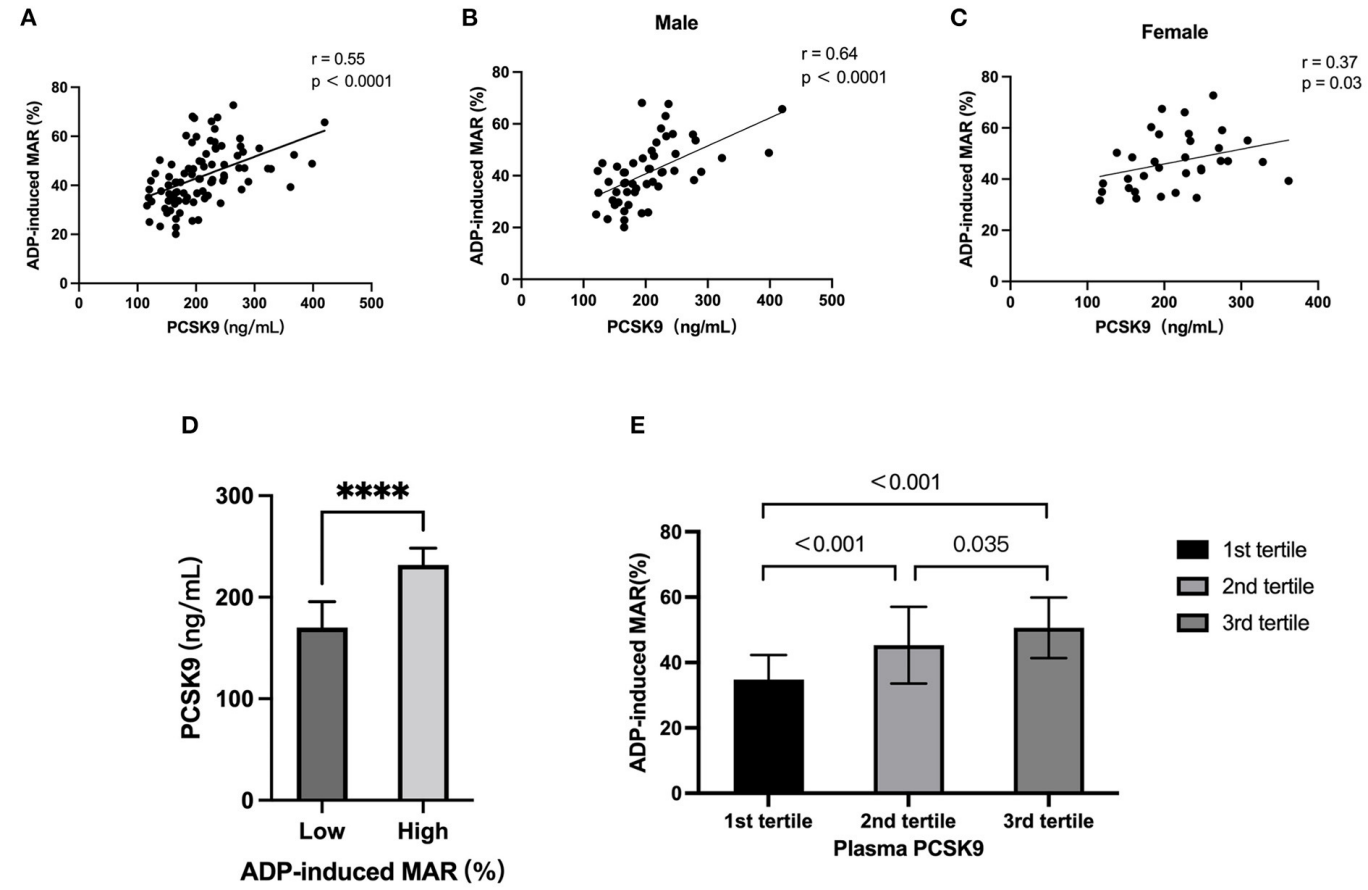
Association between proprotein convertase subtilisin/kexin type 9 (PCSK9) levels and ADP-induced platelet maximal aggregation rate (MAR). **(A)** Univariate linear correlation analysis of serum PCSK9 with ADP-induced MAR. **(B,C)** The correlation of serum PCSK9 with ADP-induced MAR was stratified by sex. **(D)** Comparison of ADP-induced MAR in subject with different PCSK9 according to tertile value. **(E)** Comparison of serum PCSK9 in subjects with ADP-induced MAR lower and higher than mean value. ****: *P* < 0.0001.

A direct linear correlation was found between increased plasma PCSK9 levels and adenosine diphosphate (ADP)-induced maximal aggregation rate (MAR) (*r* = 0.555, *p*<0.001; Figure 5A). This correlation was also observed in both female and male subgroups despite difference in ADP-induced MAR between sex (Figures 5B,C).

The distribution of ADP-induced MAR between subgroups of subjects with different PCSK9 value exhibited a trend of increment when PCSK9 increase (Supplementary Figure 2A). Vice versa, the distribution of PCSK9 in subjects with higher ADP-induced MAR above the mean value compared to subjects with low ADP-induced MAR showed a correlation with high PCSK9 level (Supplementary Figure 2B).

When assessed according to tertile values of PCSK9, there was a significant increase in ADP-induced MAR in the 2nd-tertile compared to 1st-tertile (43.5 ± 11.7% vs. 35.0 ± 7.6%; *p*<0.001). In addition, an increase of ADP-induced MAR was observed in the 3rd-tertile compared to 2nd-tertile (48.7 ± 9.3% vs. 43.5 ± 11.7%; *p* = 0.035; Figure 5D). On the other hand, subjects with high ADP-induced MAR had significantly higher plasma level of PCSK9 (Figure 5E).

Plasma PCSK9 level was not associated with AA-induced platelet aggregation in the correlation analysis. In addition, AA-induced platelet MAR was not different according to tertile value of PCSK9. Plasma PCSK9 concentration in subjects with high ADP-induced MAR was similar compared to those with low ADP-induced MAR (Supplementary Figure 3).

### Univariate and multivariate linear regression analysis

In univariate linear regression analysis, PCSK9 (*β* = 0.089, *p*<0,001), TC (*β* = 3.254, *p* =0.028), LDL-C (*β* = 4.779, *p* = 0.009), PLT (*β* = 0.041, *p* = 0.005), and smoking (*β* = −8.832, *p* = 0.016) were factors that could significantly predict the value of ADP-induced MAR (Table 1). In multivariate regression analysis, only parameters of covariates that were retained in the model during stepwise elimination procedure are included in model 2. PCSK9 (*β* = 0.09, *p* = 0.001), LDL-C (*β* = 4.81, *p* = 0.046), and PLT (*β* = 0.05, *p* = 0.005) were found to predict ADP-induced MAR. The adjusted R^2^ of the multivariate model was 0.284, *p*<0.001 (Table 2).

**Table 1:** Univariate linear regression analysis regarding the association of ADP-induced MAR and characteristics.

**Variate**	**Univariate**	
	**β**	***p*** **-value**
PCSK9	0.089	<0.001
TC	3.254	0.028
LDL-C	4.779	0.009
PLT	0.041	0.005
Sex	−4.026	0.096
Smoking	−8.832	0.016

ADP, Adenosine diphosphate; PCSK9, Proprotein Convertase Subtilisin/Kexin Type 9; MAR, maximal aggregation rate; TC, total cholesterol; LDL-C, low density lipoprotein-cholesterol; PLT, platelet.

**Table 2:** Multivariate linear regression analysis of the association of ADP-induced MAR and variables.

**Variables**	**Model 1**		**Model 2**	
	**(adjusted R**^**2**^ **0.27**,		**(adjusted R**^**2**^ **0.284**,	
	***p*** = **0.002)**		***p*** = **0.001)**	
	**β**	***p*** **-value**	**β**	***p*** **-value**
PCSK9	0.08	0.004	0.09	0.001
TC	−2.92	0.591	/	/
LDL-C	7.58	0.236	4.81	0.046
PLT	0.04	0.073	0.05	0.005
Sex	−0.63	0.83	/	/
Smoking	−4.73	0.262	/	/

ADP, Adenosine diphosphate; PSCK9, Proprotein Convertase Subtilisin/kexin Type 9; MAR, maximal aggregation rate; TC, total cholesterol; LDL-C, low density lipoprotein-cholesterol; PLT, platelet.

## Discussion

The role of PCSK9 in altering plasma LDL-C *via* PCSK9-LDLR axis has been well established; recent studies have suggested a possible role of PCSK9 in regulating platelet function. Our study is the first to confirm an independent and positive correlation between PCSK9 level and platelet reactivity in populations without established CVD, who did not take statin or antiplatelet agents.

Platelet reactivity refers to the degree of the response of blood platelets to an external stimulus. Agonists such as ADP and collagen activate platelets by binding to specific receptors that are presented on the platelet surface membrane. Platelet activation leads to an increase of intra-cytoplasmatic concentration of calcium and platelet shape change, enabling platelets to interact with each other and aggregate (18). On the other hand, platelet activation induces conformational changes in GPIIb/IIIa that transform it into its fibrinogen binding form through activating phospholipase Cβ (PLCβ) or phospholipase Cγ (PLCγ) (19). The receptor-bound fibrinogen connects two GPIIb/IIIa molecules on nearby platelets. This process is the final common pathway of agonist-induced platelet aggregation (20).

Platelet aggregation is modified by activating and inactivating biomolecules and conditions. Some components circulating in the blood can potentiate the activation process in the presence of a strong agonist. For example, adrenaline lowers cytosolic cAMP levels and augments platelet activation; insulin-like growth factor I and thrombopoietin enhance platelet activation *via* phosphatidylinositol 3-kinase (PI3K) signaling pathway (21). Diabetes mellitus and states of increased vascular stress might increase the responsiveness of platelets to agonists (22). On the other hand, bioactive mediators released from endothelial cells, such as prostaglandin I_2_ (PGI_2_), prostaglandin E_2_ (PGE2), and nitric oxide (NO), were negative platelet-priming substances (23). A study indicated that polyunsaturated fatty acid products of 12-lipoxygenase can also hamper platelet activation (24).

Measurement of platelet aggregation in platelet-rich plasma using light transmission aggregometry (LTA) is considered the “gold standard” for measurement but is complex and technically demanding. Newer approaches to measuring platelet aggregation uses impedance aggregometry, which is based on the measurement of the electrical resistance between two electrodes immersed in stirred whole blood. As platelets aggregate and bind to the electrodes, there is a change in electrical impedance that corresponds to the degree of aggregation that has occurred. Therefore, it is deemed to be more physiological than studies performed in platelet-rich plasma (25).

ADP is one of the major components released from activated platelets and it acts as an agonist at two platelet purinergic G-protein coupled receptors—the Gq-coupled P2Y_1_ and Gi-coupled P2Y_12_ receptor. While P2Y_1_ activation is responsible for intracellular calcium mobilization, shape change, and initiation of aggregation, the P2Y_12_ receptor is responsible for the completion of the aggregation to ADP (26). In the present study we found that ADP-induced platelet aggregation was associated with PLT, PCSK9, and LDL-C in multivariate regression model.

In multivariate regression analysis, ADP-induced platelet aggregation was associated with PLT, which has been described previously in patients after recent coronary stent-implantation and on dual-antiplatelet therapy (27).

Previous studies have found a correlation between plasma PCSK9 and platelet reactivity in patients with coronary artery disease and hypercholesterolemia. In a cohort of stable coronary artery disease patients, plasma PCSK9 levels were positively correlated with the platelet count and plateletcrit (13). The PCSK9-REACT study found that, in patients with a recent acute coronary syndrome (ACS) undergoing percutaneous coronary intervention and receiving P2Y12 inhibitor, there was a direct association between PCSK9 plasma level and high-on-treatment platelet reactivity (14). In patients with hypercholesterolemia who received background statin and acetyl salicylic acid therapy, platelet function parameters were significantly reduced after 12 months of treatment with the monoclonal antibody (mAb) anti-PCSK9 alirocumab or evolocumab (28). However, in these studies, use of statin and aspirin will affect both the plasma PCSK9 level and platelet activity (15, 16). In our study, a significant association of PCSK9 with increased ADP-induced MAR was detected in healthy subjects without statin and antiplatelets. Importantly, this association was significant even after adjusting for other covariates.

Similar to a previous study in CAD, platelet reactivity correlated with plasma LDL-C level in the present study (29, 30). Hypercholesterolemia may influence platelet reactivity through several mechanisms. 1) Formation of ox-LDL, which was induced by high LDL-C, could activate platelets by binding with CD36 and LOX-1 receptors (31–33). 2) Cholesterol incorporation in plasma membranes induces platelet hypersensitivity to stimuli, whereas its depletion strikingly reduces platelet reactivity (34–36). However, PCSK9 correlated with platelet reactivity after adjusting LDL-C level in multivariate linear regression in our study, suggesting PCSK9 may affect platelet activity through a lipid-lowering independent mechanism. A recent study found PCSK9 can directly enhance agonist-induced platelet activation by binding to platelet CD36 and thus activating Src kinase and MAPK-extracellular signal-regulated kinase 5 and c-Jun N terminal kinase, increasing the generation of reactive oxygen species and activating the p38MAPK/cytosolic phospholipase A2/cyclooxygenase-1/thromboxane A2 signaling pathway (10).

Another finding of this study is AA-induced platelet aggregation was not correlated to plasma PCSK9. Arachidonic acid is derived from membrane phospholipids through phospholipase A2 (PLA2). AA is transformed into prostaglandin G2 and prostaglandin H2 by cyclooxygenase-1(COX-1), then transformed into TXA2, which is a strong activator of platelets. Our finding suggests PCSK9 does not affect PLA2/Cox-1/TXA2 pathway.

The limitation of the present study is its cross-sectional nature. The findings of our study could only indicate associations, not causality. Another limitation is the relatively small sample size of the population.

## Conclusions

In summary, PCSK9 levels are associated with platelet activation in subjects not taking statins or antiplatelet agents. Subjects with increased concentration of PCSK9 have significantly higher platelet activation. The finding of this study provides additional evidence of the correlation between PCSK9 level and platelet activation beyond CVD patients. Future studies are warranted to further elucidate the role of PCSK9 as a risk factor for ASCVD.

## Data availability statement

The raw data supporting the conclusions of this article will be made available by the authors, without undue reservation.

## Ethics statement

The studies involving human participants were reviewed and approved by the Ethics Committee of Second Xiangya Hospital on research on humans. The patients/participants provided their written informed consent to participate in this study.

## Author contributions

SW analyzed the result and wrote the manuscript. DF and HL collected the datasets. DP conceived the study and revised the manuscript. All authors contributed to the article and approved the submitted version.

## Funding

This work was supported by grants from the National Nature Science Foundation Youth Project (81600359) and the National Nature Science Foundation General Project (81870336).

## Conflict of interest

The authors declare that the research was conducted in the absence of any commercial or financial relationships that could be construed as a potential conflict of interest.

## Publisher's note

All claims expressed in this article are solely those of the authors and do not necessarily represent those of their affiliated organizations, or those of the publisher, the editors and the reviewers. Any product that may be evaluated in this article, or claim that may be made by its manufacturer, is not guaranteed or endorsed by the publisher.

## References

[1] Berger JS Becker RC Kuhn C Helms MJ Ortel TL Williams R. Hyperreactive platelet phenotypes: relationship to altered serotonin transporter number, transport kinetics and intrinsic response to adrenergic co-stimulation. Thromb Haemost. (2013) 109:85–92. 10.1160/TH12-03-0202 23223800 PMC3582386

[2] Kondkar AA Bray MS Leal SM Nagalla S Liu DJ Jin Y. VAMP8/endobrevin is overexpressed in hyperreactive human platelets: suggested role for platelet microRNA. J Thromb Haemost. (2010) 8:369–78. 10.1111/j.1538-7836.2009.03700.x 19943878 PMC3312605

[3] Montenont E Echagarruga C Allen N Araldi E Suarez Y Berger JS. Platelet WDR1 suppresses platelet activity and is associated with cardiovascular disease. Blood. (2016) 128:2033–42. 10.1182/blood-2016-03-703157 27609643 PMC5073182

[4] Sharma G Berger JS. Platelet activity and cardiovascular risk in apparently healthy individuals: a review of the data. J Thromb Thrombolysis. (2011) 32:201–8. 10.1007/s11239-011-0590-9 21562837

[5] Trip MD Cats VM van Capelle FJ Vreeken J. Platelet hyperreactivity and prognosis in survivors of myocardial infarction. N Engl J Med. (1990) 322:1549–54. 10.1056/NEJM199005313222201 2336086

[6] Tofler GH Brezinski D Schafer AI Czeisler CA Rutherford JD Willich SN. Concurrent morning increase in platelet aggregability and the risk of myocardial infarction and sudden cardiac death. N Engl J Med. (1987) 316:1514–8. 10.1056/NEJM198706113162405 3587281

[7] Fuentes F Palomo I Fuentes E. Platelet oxidative stress as a novel target of cardiovascular risk in frail older people. Vascul Pharmacol. (2017) 93-95:14–9. 10.1016/j.vph.2017.07.003 28705733

[8] El Haouari M. Platelet oxidative stress and its relationship with cardiovascular diseases in type 2 diabetes mellitus patients. Curr Med Chem. (2019) 26:4145–65. 10.2174/0929867324666171005114456 28982316

[9] Wang N Tall AR. Cholesterol in platelet biogenesis and activation. Blood. (2016) 127:1949–53. 10.1182/blood-2016-01-631259 26929273 PMC4841038

[10] Qi Z Hu L Zhang J Yang W Liu X Jia D. PCSK9 (proprotein convertase subtilisin/kexin 9) enhances platelet activation, thrombosis, and myocardial infarct expansion by binding to platelet CD36. Circulation. (2021) 143:45–61. 10.1161/CIRCULATIONAHA.120.046290 32988222

[11] Leander K Malarstig A Van't Hooft FM Hyde C Hellenius ML Troutt JS. Circulating proprotein convertase subtilisin/kexin type 9 (PCSK9) predicts future risk of cardiovascular events independently of established risk factors. Circulation. (2016) 133:1230–9. 10.1161/CIRCULATIONAHA.115.018531 26896437

[12] Xie W Liu J Wang W Wang M Qi Y Zhao F. Association between plasma PCSK9 levels and 10-year progression of carotid atherosclerosis beyond LDL-C: a cohort study. Int J Cardiol. (2016) 215:293–8. 10.1016/j.ijcard.2016.04.103 27128549

[13] Li S Zhu CG Guo YL Xu RX Zhang Y Sun J. The relationship between the plasma PCSK9 levels and platelet indices in patients with stable coronary artery disease. J Atheroscler Thromb. (2015) 22:76–84. 10.5551/jat.25841 25185779

[14] Navarese EP Kolodziejczak M Winter MP Alimohammadi A Lang IM Buffon A. Association of PCSK9 with platelet reactivity in patients with acute coronary syndrome treated with prasugrel or ticagrelor: the PCSK9-REACT study. Int J Cardiol. (2017) 227:644–9. 10.1016/j.ijcard.2016.10.084 27810295

[15] Sahebkar A Simental-Mendia LE Guerrero-Romero F Golledge J Watts GF. Effect of statin therapy on plasma proprotein convertase subtilisin kexin 9 (PCSK9) concentrations: a systematic review and meta-analysis of clinical trials. Diabetes Obes Metab. (2015) 17:1042–55. 10.1111/dom.12536 26183252

[16] Nenna A Nappi F Lusini M Satriano UM Schiliro D Spadaccio C. Effect of statins on platelet activation and function: from molecular pathways to clinical effects. Biomed Res Int. (2021) 2021:6661847. 10.1155/2021/6661847 33564680 PMC7850835

[17] Camera M Rossetti L Barbieri SS Zanotti I Canciani B Trabattoni D. PCSK9 as a positive modulator of platelet activation. J Am Coll Cardiol. (2018) 71:952–4. 10.1016/j.jacc.2017.11.069 29471945

[18] Varga-Szabo D Braun A Nieswandt B. Calcium signaling in platelets. J Thromb Haemost. (2009) 7:1057–66. 10.1111/j.1538-7836.2009.03455.x 19422456

[19] Varga-Szabo D Pleines I Nieswandt B. Cell adhesion mechanisms in platelets. Arterioscler Thromb Vasc Biol. (2008) 28:403–12. 10.1161/ATVBAHA.107.150474 18174460

[20] Koltai K Kesmarky G Feher G Tibold A Toth K. Platelet aggregometry testing: molecular mechanisms, techniques and clinical implications. Int J Mol Sci. (2017) 18:1803. 10.3390/ijms18081803 28820484 PMC5578190

[21] van der Meijden PEJ Heemskerk JWM. Platelet biology and functions: new concepts and clinical perspectives. Nat Rev Cardiol. (2019) 16:166–79. 10.1038/s41569-018-0110-0 30429532

[22] Kraakman MJ Lee MK Al-Sharea A Dragoljevic D Barrett TJ Montenont E. Neutrophil-derived S100 calcium-binding proteins A8/A9 promote reticulated thrombocytosis and atherogenesis in diabetes. J Clin Invest. (2017) 127:2133–47. 10.1172/JCI92450 28504650 PMC5451242

[23] Naseem KM Roberts W. Nitric oxide at a glance. Platelets. (2011) 22:148–52. 10.3109/09537104.2010.522629 21050056

[24] Tourdot BE Adili R Isingizwe ZR Ebrahem M Freedman JC Holman TR. 12-HETrE inhibits platelet reactivity and thrombosis in part through the prostacyclin receptor. Blood Adv. (2017) 1:1124–31. 10.1182/bloodadvances.2017006155 29296755 PMC5728320

[25] Algahtani M Heptinstall S. Novel strategies for assessing platelet reactivity. Future Cardiol. (2017) 13:33–47. 10.2217/fca-2016-0054 27990840

[26] Gremmel T Frelinger AL III Michelson AD. Platelet physiology. Semin Thromb Hemost. (2016) 42:191–204. 10.1055/s-0035-1564835 26926581

[27] Olivier CB Meyer M Bauer H Schnabel K Weik P Zhou Q. The ratio of ADP- to TRAP-induced platelet aggregation quantifies P2Y12-dependent platelet inhibition independently of the platelet count. PLoS ONE. (2016) 11:e0149053. 10.1371/journal.pone.0149053 26885820 PMC4757031

[28] Barale C Bonomo K Frascaroli C Morotti A Guerrasio A Cavalot F. Platelet function and activation markers in primary hypercholesterolemia treated with anti-PCSK9 monoclonal antibody: a 12-month follow-up. Nutr Metab Cardiovasc Dis. (2020) 30:282–91. 10.1016/j.numecd.2019.09.012 31653513

[29] Petersen-Uribe A Kremser M Rohlfing AK Castor T Kolb K Dicenta V. Platelet-derived PCSK9 is associated with LDL metabolism and modulates atherothrombotic mechanisms in coronary artery disease. Int J Mol Sci. (2021) 22:1179. 10.3390/ijms222011179 34681838 PMC8538687

[30] DiMinno G Silver MJ Cerbone AM Rainone A Postiglione A Mancini M. Increased fibrinogen binding to platelets from patients with familial hypercholesterolemia. Arteriosclerosis. (1986) 6:203–11. 10.1161/01.ATV.6.2.203 3954674

[31] Magwenzi S Woodward C Wraith KS Aburima A Raslan Z Jones H. Oxidized LDL activates blood platelets through CD36/NOX2-mediated inhibition of the cGMP/protein kinase G signaling cascade. Blood. (2015) 125:2693–703. 10.1182/blood-2014-05-574491 25710879 PMC4408294

[32] Chen K Febbraio M Li W Silverstein RL. A specific CD36-dependent signaling pathway is required for platelet activation by oxidized low-density lipoprotein. Circ Res. (2008) 102:1512–9. 10.1161/CIRCRESAHA.108.172064 18497330 PMC2749986

[33] Hofmann A Brunssen C Morawietz H. Contribution of lectin-like oxidized low-density lipoprotein receptor-1 and LOX-1 modulating compounds to vascular diseases. Vascul Pharmacol. (2017) 19:S1537-1891(17)30171-4. 10.1016/j.vph.2017.10.002 29056472

[34] Shattil SJ Anaya-Galindo R Bennett J Colman RW Cooper RA. Platelet hypersensitivity induced by cholesterol incorporation. J Clin Invest. (1975) 55:636–43. 10.1172/JCI107971 1117069 PMC301792

[35] Tomizuka T Yamamoto K Hirai A Tamura Y Yoshida S. Hypersensitivity to thromboxane A2 in cholesterol-rich human platelets. Thromb Haemost. (1990) 64:594–9. 10.1055/s-0038-1647364 2150728

[36] Korporaal SJ Meurs I Hauer AD Hildebrand RB Hoekstra M Cate HT. Deletion of the high-density lipoprotein receptor scavenger receptor BI in mice modulates thrombosis susceptibility and indirectly affects platelet function by elevation of plasma free cholesterol. Arterioscler Thromb Vasc Biol. (2011) 31:34–42. 10.1161/ATVBAHA.110.210252 21051668

