# Supplementary material for: Independent association of PCSK9 with platelet reactivity in subjects without statin or antiplatelet agents
Source: Front Cardiovasc Med. 2022 Oct 17;9:934914. doi: 10.3389/fcvm.2022.934914 (PMC9618652; doi:10.3389/fcvm.2022.934914)
Supplement: Supplementary file 1 [file Data_Sheet_1.docx]

Supplementary Material

# 1. Supplementary Figure 1.


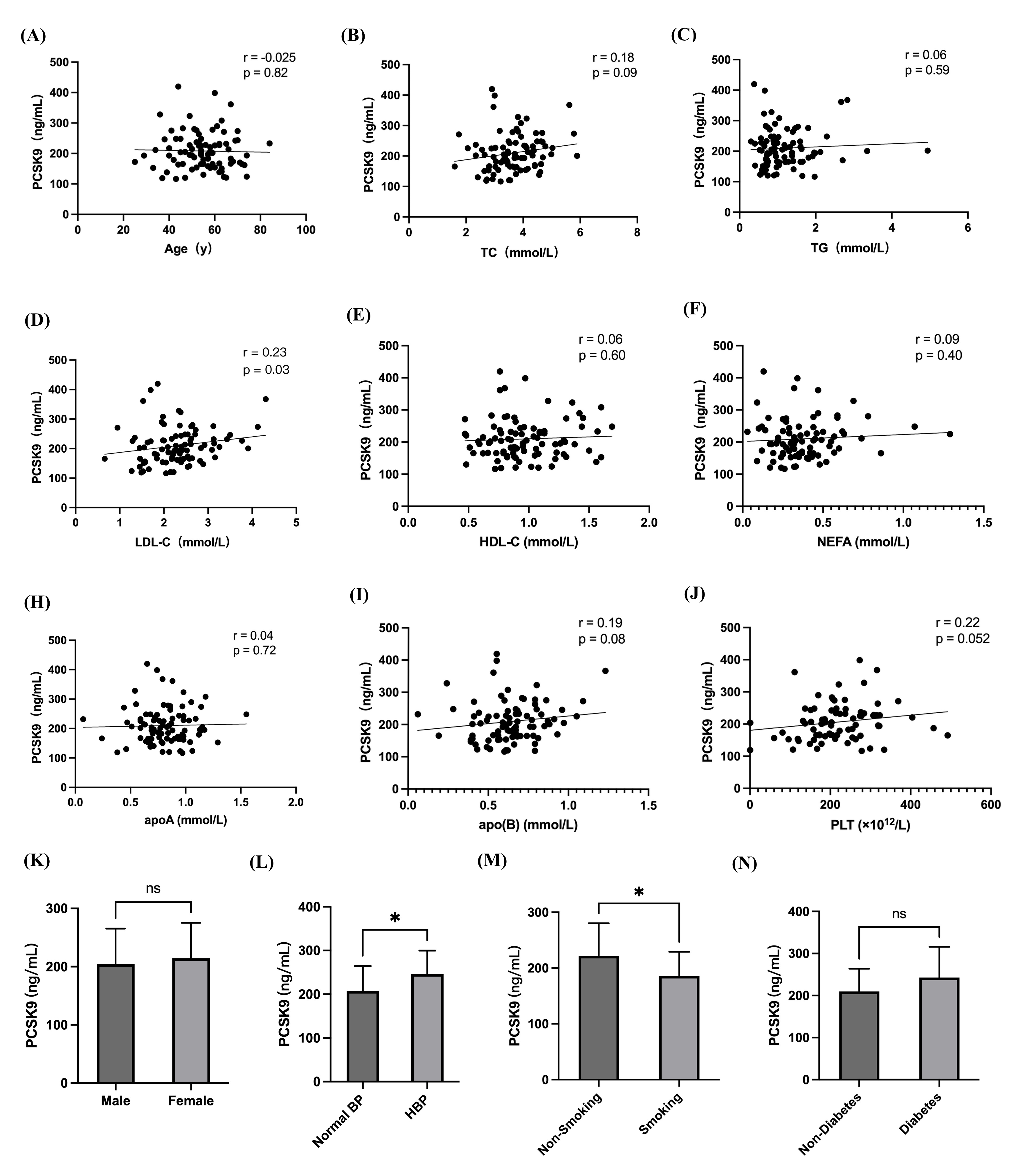


**Supplementary Figure 1. Correlation between plasma PCSK9 concentration and baseline characteristics.** Univariate linear correlation analysis of subjects characteristics including age (A), TC (B), TG (C), LDL-C (D), HDL-C (E), NEFA (F), apoA (G), apoB (H), and PLT (I) with plasma PCSK9. Plasma PCSK9 concentration stratified by Sex (K), Hypertension (L), Smoking (M) and Diabetes (N).

# 2. Supplementary Figure 2.


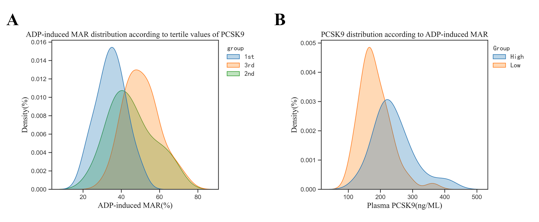


**Supplementary Figure 2.** **Distribution of platelet reactivity and PCSK9.** (A) Distribution of ADP-induced MAR according to tertile values of PCSK9. (B) Distribution of PCSK9 according to high versus low ADP-induced MAR.

## 3. Supplementary Figure 3


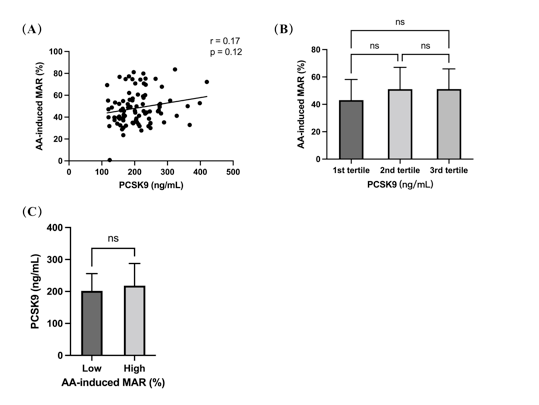


**Supplementary Figure 3. Association between proprotein convertase subtilisin/kexin type 9 (PCSK9) levels and AA-induced platelet maximal aggregation rate.** (A) Univariate linear correlation analysis of serum PCSK9 with AA-induced platelet maximal aggregation rate (MAR). (B) Comparison of AA-induced platelet maximal aggregation rate in subject with different PCSK9 according to tertile value. (C). Comparison of serum PCSK9 in subjects with AA-induced maximal platelet aggregation rate (MAR) lower and higher than mean value.

# 4. Supplementary Table 1. Characteristics of population.

|  | Total  (n=89) | First Tertile PCSK9  (149[116,172]ng/mL)  (n=29) | Second Tertile PCSK9  (200[173,227]ng/mL)  (n=30) | Third Tertile PCSK9  (277[227,419]ng/mL)  (n=30) | *p*-value |
| --- | --- | --- | --- | --- | --- |
| Age, years | 55±11 | 57±11 | 55±8 | 56±12 | 0.58 |
| Male, n(%) | 53 (60) | 20 (69) | 19 (68) | 14 (48) | 0.22 |
| Hypertension, n(%) | 13 (14) | 1 (6) | 3 (17) | 9 (35) | 0.059 |
| Diabetes, n(%) | 8 (9) | 2 (11) | 1 (6) | 5 (20) | 0.51 |
| Smoking, n(%) | 13 (15) | 7 (39) | 4 (22) | 2 (8) | 0.04^*^ |
| TC, mmol/L | 3.60±0.82 | 3.40±0.72 | 3.50±0.76 | 3.80±0.84 | 0.07 |
| TG, mmol/L | 0.95 (0.71, 1.42) | 0.90 (0.71, 1.35) | 1.15 (0.77, 1.68) | 0.94 (0.71, 1.42) | 0.32 |
| HDL-C, mmol/L | 0.96±0.29 | 0.97±0.27 | 0.86±0.25 | 1.06±0.31 | 0.20 |
| LDL-C, mmol/L | 2.28±0.67 | 2.06±0.58 | 2.26±0.58 | 2.39±0.69 | 0.049^*^ |
| NEFA, mmol/L | 0.34 (0.25, 0.47) | 0.35 (0.25, 0.43) | 0.34 (0.23, 0.48) | 0.29 (0.20, 0.47) | 0.40 |
| Apo(A), mmol/L | 0.80±0.23 | 0.78±0.21 | 0.78±0.18 | 0.82±0.28 | 0.62 |
| Apo(B), mmol/L | 0.64±0.20 | 0.59±0.17 | 0.66±0.16 | 0.63±0.22 | 0.18 |
| PLT, ×10^12^/L | 221±88 | 197±95 | 230±100 | 235±64 | 0.21 |

Abbreviations: TC, total cholesterol; TG, triglyceride; HDL, high-density lipoprotein-cholesterol; LDL-C, low density lipoprotein-cholesterol; NEFA, nonesterified fatty acid; apo(A), liprotein A, apo(B), lipoprotein B; PLT, platelet.
